# RNAi Factors are Present and Active in Human Cell Nuclei

**DOI:** 10.1016/j.celrep.2013.12.013

**Published:** 2014-01-02

**Authors:** Keith T. Gagnon, Liande Li, Yongjun Chu, Bethany A. Janowski, David R. Corey

**Affiliations:** Departments of Pharmacology and Biochemistry, University of Texas Southwestern Medical Center, Dallas, Texas, 75390-9041

## Abstract

RNAi is widely appreciated as a powerful regulator of mRNA translation in the cytoplasm of mammalian cells. However, the presence and activity of RNAi factors in the mammalian nucleus has been the subject of considerable debate. Here we show that Argonaute-2 (Ago2) and RNAi factors Dicer, TRBP and TRNC6A/GW182 are in the human nucleus and associate together in multi-protein complexes. Small RNAs can silence nuclear RNA and guide site-specific cleavage of the targeted RNA, demonstrating that RNAi can function in the human nucleus. Nuclear Dicer is active and miRNAs are bound to nuclear Ago2, consistent with the existence of nuclear miRNA pathways. Notably, we do not detect loading of duplex small RNAs in nuclear extracts and known loading factors are absent. These results extend RNAi into the mammalian nucleus and suggest that regulation of RNAi via small RNA loading of Ago2 differs between the cytoplasm and the nucleus.

## Introduction

Since the discovery of mammalian RNA interference (RNAi) (Elbashir et al., 2001), over 50,000 reports have described the use of small interfering RNAs (siRNAs). Almost all of these studies have assumed that the regulation of RNAi and its silencing activity occurs in the cytoplasm (Gurtan and Sharp, 2013). Whether RNAi can function in the mammalian nucleus and regulate processes like transcription or splicing has remained unclear (Castel and Martienssen, 2013; Harel-Bellan et al., 2013). Likewise, what role the nuclear compartment might play in the regulation of RNAi pathways is unknown. These uncertainties have significantly hampered investigation of nuclear RNA biology and the development of nuclear RNAi as a laboratory tool and potential therapeutic.

The assumption that mammalian RNAi is confined to the cytoplasm has been supported by reports that siRNAs cannot silence introns (Vickers et al., 2003; Zeng and Cullen, 2002). In addition, microscopy has shown a cytoplasmic distribution of RNAi factors, such as argonaute 2 (Ago2), to p-bodies and the endoplasmic reticulum (ER) (Ikeda et al., 2006; Stalder et al., 2013). Some laboratories, however, have suggested that Ago2 and other RNAi factors can be found in the nucleus (Ando et al., 2011; Chu et al., 2010; Doyle et al., 2013; Ohrt et al., 2012; Rudel et al., 2008; Till et al., 2007; Weinmann et al., 2009). siRNAs have been reported to silence the nuclear enriched RNAs 7SK and U6 (Ohrt et al., 2008; Robb et al., 2005). Although nuclear RNAi activity and localization of RNAi factors to the nucleus have been reported previously, questions about the purity of cell extracts (Holding, 2005), the resolution of localization studies, and nucleocytoplasmic transport of the RNA targets and products of RNAi have kept nuclear RNAi a controversial subject.

MicroRNAs (miRNAs) enter the RNAi pathway by binding Ago proteins (Gurtan and Sharp, 2013). In the cytoplasm, miRNAs guide Ago proteins to 3′ untranslated regions and destabilize or inhibit translation of mRNAs (Bartel, 2009; Gurtan and Sharp, 2013; Valencia-Sanchez et al., 2006). miRNAs have also been found in the nucleus (Jeffries et al., 2011; Katahira and Yoneda, 2011; Liao et al., 2010) but their biological roles are unknown. Both synthetic siRNAs and microRNAs have been shown to induce changes in splicing (Allo et al., 2009; Liu et al., 2012) and transcription (Janowski et al., 2007; Li et al., 2006; Matsui et al., 2013; Morris et al., 2004). However, the mechanisms mediating these processes remain controversial, due in part to the debate over the presence and activity of nuclear RNAi factors.

During cytoplasmic RNAi, small RNA loads into the RNA induced silencing complex (RISC), the complex recognizes a complementary RNA target, and target cleavage can occur at a specific site (Wilson and Doudna, 2013). Several factors have been implicated in the loading of small RNAs into Ago proteins (programming) and the maturation of RISC in human cells. These include the protein folding chaperones Hsp90 and Hsc70 (Iwasaki et al., 2010) and the component 3 promoter of RNAi (C3PO) complex composed of Translin and TRAX (Ye et al., 2011). Hsp90/Hsc70 are implicated in chaperone-like mechanisms that may open Ago proteins to accommodate the initial binding of a duplex RNA (Iwasaki et al., 2010). In addition, Hsp90 chaperone activity in RNAi programming and RISC maturation has been shown to be dependent on the presence of co-chaperones, including Aha1, FKBP4/5, Cdc37 and p23 (Martinez et al., 2013; Pare et al., 2013). C3PO possesses single-strand nuclease activity and has been shown to accelerate passenger strand RNA removal from Ago to mature the RISC complex (Liu et al., 2009; Ye et al., 2011).

Ago2 binds small duplex RNA and forms the core of RISC (Hammond et al., 2000; MacRae et al., 2008; Wilson and Doudna, 2013). Other key players involved in RISC include the pre-miRNA processing enzyme Dicer, the TAR RNA-binding protein (TRBP), and TNRC6A (GW182 homolog) (Daniels and Gatignol, 2012; Lazzaretti et al., 2009; Ma et al., 2012; MacRae et al., 2008). Ago-RISC complexes recognize RNAs complementary to the guide strand (Hammond et al., 2000). When the guide strand is fully complementary to target RNA, Ago2 can catalyze site-specific phosphodiester bond cleavage (“slicer” activity) (Liu et al., 2004; Meister et al., 2004; Wang et al., 2008).

To resolve the controversy over mammalian nuclear RNAi, we investigated the localization, interaction, and activity of known RNAi factors in human cell nuclei. Here we show that Ago2 and other RNAi factors are present in the nucleus and can associate in multi-protein complexes. Small RNAs in complex with Ago2 can silence nuclear RNA and induce site-specific cleavage. Nuclear Dicer is catalytically active and miRNAs are bound to nuclear Ago2. In contrast, we did not detect loading of duplex RNA in nuclei and most RISC loading factors are absent. These results place the protein machinery necessary for RNAi recognition inside the mammalian cell nucleus but suggest key differences between nuclear and cytoplasmic RNAi.

## Results

### RNAi Factors are Present in Human Cell Nuclei

We began our study by using HeLa cells to examine the localization of Ago2, the catalytic core of RNAi (Liu et al., 2004). We used wide-field immuno-epifluorescence microscopy with blind deconvolution because the technique is ideal for rapid and high sensitivity 3-D imaging for thin specimens such as cells in monolayer culture (Shaw, 2006). In some cases, we also used confocal immunofluorescence microscopy for comparison. The success of immuno-fluorescence often depends on conditions like fixation and permeabilization, antibody binding, and cell type (Katikireddy and O'Sullivan, 2011). To improve detection of nuclear proteins, we used protocols designed to facilitate entry of antibody into the nucleus (Spector, 2011).

**Figure 1:**
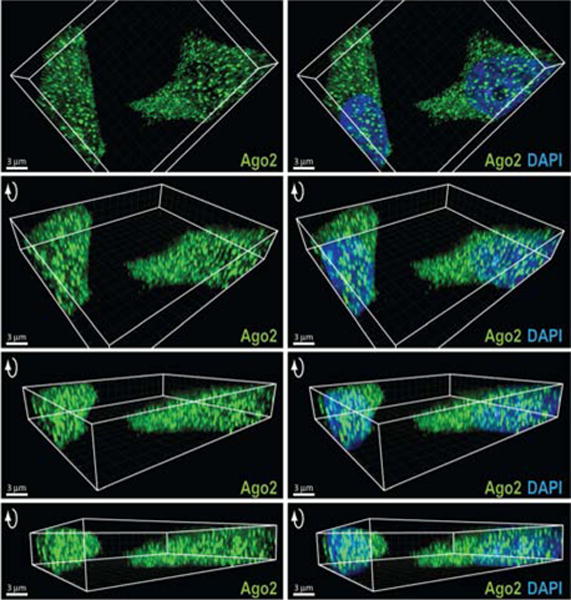
Microscopy reveals Ago2 in human cell nuclei Immuno-fluorescence microscopy of endogenous Ago2 in HeLa cells. Z-sections are stacked (6 μm), projected three-dimensionally, and rotated to highlight nuclear staining. Scale bar = 3 μm.

Our microscopy revealed a substantial amount of Ago2 in the nucleus in addition to the expected distribution within the cytoplasm. Images of slices through several micrometer thick sections along the z-axis, combined with 3-D rendering of composite focal sections, revealed Ago2 within the nuclear compartment (Figure 1; Movie S1). The application of two additional antibodies against human Ago2 produced similar results (Figure S1A-C), confirming that nuclear visualization was not due to off-target immunoreactivity. We also observed Ago2 in the nuclei of T47D breast cancer cells and fibroblast cells (Figure S1D-F), indicating that nuclear Ago2 is not cell-type specific. Confocal microscopy confirmed nuclear localization of Ago2 (Figure S1G-H). We also used wide-field immunofluorescence microscopy to test localization of Dicer, TRBP and TNRC6A and observed staining in the nucleus as well as in the cytoplasm (Figure S1I-K). These results using different microscopy platforms, cell lines, and detection reagents suggest the nuclear presence of the protein machinery that enables RNAi.

**Figure 2:**
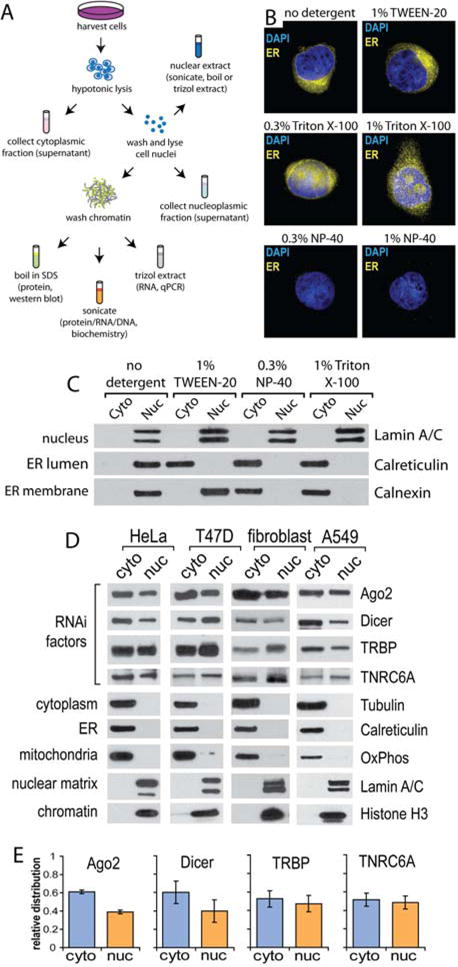
RNAi factors are present in nuclear extracts (**A**) Schematic of cellular fractionation protocol. (**B**) Fluorescent imaging of intact HeLa nuclei after isolation with buffers containing different detergents. ER membrane is stained yellow with ER Tracker dye, DAPI staining of nuclei. (**C**) Western blot analysis of cytoplasmic and nuclear fractions prepared with buffers containing different detergents. Calreticulin is a marker for the ER lumen. Calnexin is a marker fro the ER membrane. (**D**) Western blot of RNAi factors and subcellular markers from cytoplasmic and nuclear fractions prepared with 0.3% NP-40. Oxphos is a marker for mitchodondria, Lamin A/C is a marker for nuclear matrix, and tubulin is a marker for cytoplam. (**E**) Quantification of RNAi factors from Western blots of HeLa, T47D, fibroblast and A549 cells shown in panel **D**. Error bar is +/- SEM.

As a second method for testing nuclear localization, we used cellular fractionation and Western blot analysis to evaluate the levels of Ago2 and other RNAi factors in the nucleus. We developed a step-wise protocol for isolating cytoplasmic, whole nuclear, nucleoplasmic (soluble nuclear), and chromatin-associated fractions from the same cell population. Fractions were used for various assays, including protein and RNA detection, chromatographic or biochemical fractionation, and enzymatic assays (Figure 2A).

RNAi factors can localize to the endoplasmic reticulum (ER) (Stalder et al., 2013). This poses a challenge for accurate assessment of localization inside of nuclei because the ER membrane is contiguous with the outer nuclear membrane (Hetzer, 2010). To ensure efficient removal of ER protein contamination, we tested detergents and conditions for preparation of nuclear extracts (Michelson and von Hagen, 2009). Nuclei were washed with buffers containing different detergents and then visualized by fluorescence microscopy using DAPI and ER-tracker, a fluorescent dye that binds the sulphonylurea receptor class of ER integral membrane proteins. Our microscopy indicated that the addition of 0.3% NP-40 was most efficient at removing ER contamination (Figure 2B). Western blots confirmed the absence of both ER lumen and ER membrane proteins from nuclear extracts (Figure 2C).

Western blot analysis of purified nuclei revealed endogenous Ago2, Dicer, TRBP, and TRNC6A in the nuclei of multiple human cell lines (Figure 2D). Protein markers for the cytoplasm, ER, and mitochondria were absent from nuclear preparations, consistent with stringent isolation of nuclei. Quantitation of Western blots revealed relative nuclear abundances ranging from 40%-50% (Figure 2E). We also observed the other human Ago variants, Ago1, Ago3, and Ago4, in our nuclear preparations (Figure S2). Combined with microscopy, our analysis of cell fractions demonstrates that the basic machinery necessary to execute RNAi is present in human cell nuclei.

### Nuclear RNAi Factors Can Stably Associate in Multi-Protein Complexes

**Figure 3:**
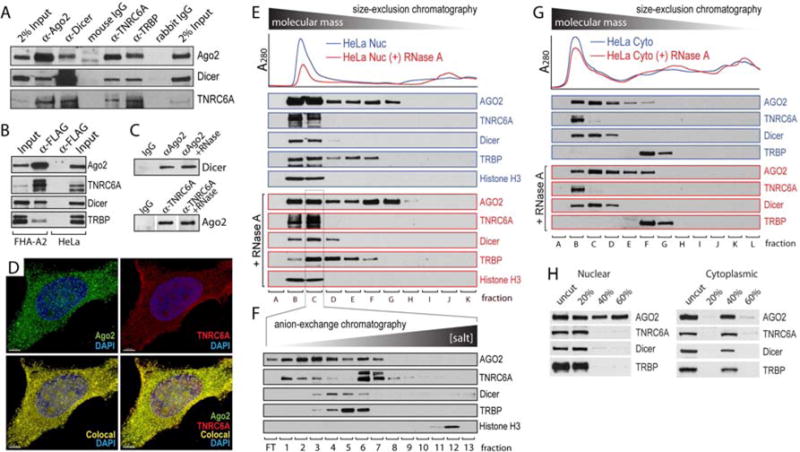
RNAi factors associate in multi-protein complexes in the nucleus (**A**) Co-immunoprecipitation (co-IP) of endogenous RNAi factor from HeLa nuclear extract. The antibodies used for the western blot detection are noted the right while the antibodies used for immunoprecipitations are on top. (**B**) Co-IP of RNAi factors from T47D cells expressing FLAG-HA-tagged Ago2 (FHA-A2). HeLa nuclear extract serves as a negative control. Input is extract prior to immunoprecipitation. (**C**) Co-IP of Ago2 with Dicer or TNRC6A from T47D nuclear extracts treated with RNase A. (**D**) Immuno-fluorescence of Ago2 and TNRC6A in HeLa cells indicates overlap and co-localization of immuno-staining. Z-stacks (3 μm of 0.1 μm slices) are projected in 3D. The co-localization channel was generated in Imaris (Bitplane). Scale bar = 5 μm. (**E**) Western analysis of fractions from separation of HeLa cell nuclear extract by size exclusion. Extracts were prepared either with our without treatment by RNAse A. Western blot antibodies are shown to the right. Sample fractions are below. Histone H3 is marker for high molecular weight chromatin. (**F**) Western analysis of fractions after anion-exchange chromatography of nuclear extract Fraction C from Figure 5E. FT, column flow-through. (**G**) Western analysis of fractions HeLa cytoplasmic extract after size exclusion chromatography. Extracts were prepared either with our without treatment with RNase A. (**H**) Effect of ammonium sulfate precipitation of RNAi factors from T47D nuclear or cytoplasmic extracts. Western blot antibodies are shown to the right and ammonium sulfate concentrations (% saturation) are shown above.

RNAi factors interact to form RISC and execute RNAi in the cytoplasm (MacRae et al., 2008; Wilson and Doudna, 2013). To determine whether nuclear RNAi factors also interact, we tested co-immunoprecipitations of nuclear Ago2, Dicer, TNRC6A and TRBP. These co-immunoprecipitations revealed a network of interactions between RNAi factors (Figure 3A).

To further support the observed association of RNAi factors with Ago2 in nuclei, we generated T47D cells stably expressing FLAG-HA-tagged Ago2 (FHA-Ago2). Immunoprecipitation of FHA-Ago2 with anti-FLAG antibody confirmed co-purification of Dicer, TNRC6A and TRBP (Figure 3B). Co-immunoprecipitation of Ago2 and Dicer or Ago2 and TNRC6A were also observed in nuclear extracts treated with RNase A, indicating that the association of RNAi factors was independent of RNA (Figure 3C).

To visualize the interaction of TNRC6A and Ago2 inside of cell nuclei, we performed immuno-fluorescence microscopy. These experiments revealed co-localization of Ago2 and TNRC6A staining within HeLa nuclei (Figure 3D; Figure S3). Co-localization of Ago2 and TNRC6A is consistent with the suggestion from our co-immunoprecipitation results that nuclear RNAi factors can form complexes.

To further characterize nuclear complexes containing RNAi factors, we separated nuclear extracts by size and charge. Fractionation by size-exclusion chromatography revealed high molecular weight complexes containing all four RNAi factors (Figure 3E). To test the involvement of RNA in complex formation, we treated extracts with RNAse A and observed that RNA was not required. When high molecular weight fractions containing RNAi factors were further separated by anion-exchange chromatography, RNAi factors continued to co-elute (Figure 3F) indicating that the complexes were sufficiently stable to survive tandem purification schemes. Taken together, these results demonstrate that nuclear RNAi factors can form stable multi-protein RISC-like complexes.

To compare nuclear and cytoplasmic RNAi protein complexes, we performed similar size exclusion chromatography with cytoplasmic extract (Figure 3G). The retention time of Ago2, Dicer, and GW182 was similar in cytoplasmic fractions compared to the nuclear fraction. TRBP, however, eluted later regardless of the presence or absence of RNase A. As an alternate test for the stability associations between RNAi factors, we fractionated nuclear and cytoplasmic extracts by adding increasing amounts of ammonium sulfate. While all four RNAi factors precipitated from nuclear extract at 20% ammonium sulfate, a 40% concentration was required for precipitation from cytoplasmic extract (Figure 3H). These results from fractionation by either chromatography or precipitation reveal the formation of RISC-like complexes, but also suggest that the exact composition of complexes in the nucleus and the stability of their association differs from that observed in the cytoplasm.

### RNAi is Active in the Nucleus

**Figure 4:**
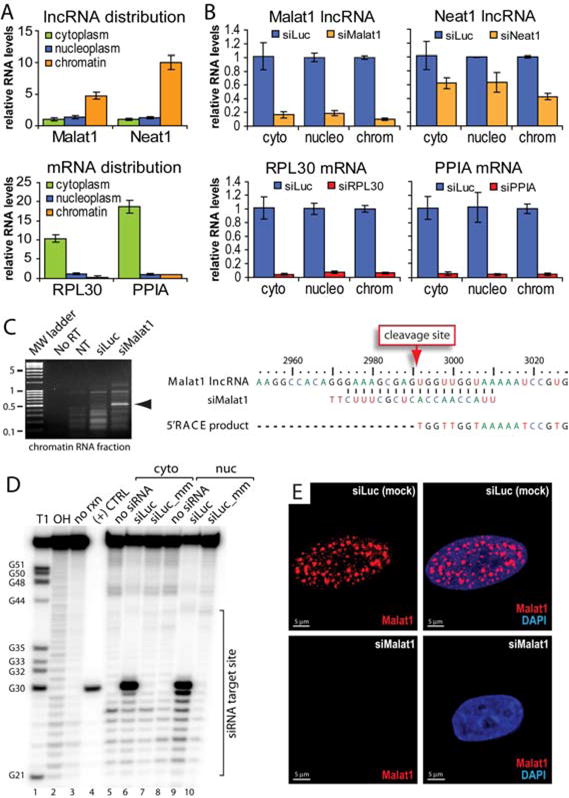
RNAi is active in the human cell nucleus (**A**) Quantification RNA distribution in HeLa sub-cellular fractions by qPCR. Error bar is SEM. (**B**) Quantification by qPCR of siRNA-mediated lncRNA and mRNA knock-down in HeLa sub-cellular fractions. Error bar is +/- SEM. (**C**) 5′ RACE to detect siRNA-mediated Ago2 cleavage of Malat1 in chromatin-associated RNA fractions. Arrow indicates specific cleavage product. (**D**) Cleavage of a 5′-radiolabeled luciferase RNA substrate by Ago2 isolated from cytoplasmic or nuclear fractions. T1, RNase T1 cleavage; OH, alkaline hydrolysis; (+) CTRL, synthetic cleavage product. The region overlapped by the siRNA is noted. (**E**) FISH showing that Malat1 speckles in HeLa cells are lost upon treatment with siMalat1 siRNA. Z-stacks are 5 μm thickness. siLuc: mock treatment. Scale bar = 5 μm.

After demonstrating that RNAi factors were present in human cell nuclei, we investigated whether they could also direct silencing of nuclear RNA substrates. We examined the silencing within cytoplasmic, nucleoplasmic, and chromatin-associated cell fractions. Our target RNAs were Malat1 and Neat1, long noncoding RNAs (lncRNAs) primarily associated with chromatin (Figure 4A) (Dodd et al., 2013). For comparison, we also targeted ribosomal protein L30 (RPL30) and peptidylprolyl isomerase A (PPIA) mRNAs, which are primarily cytoplasmic.

We treated cells with siRNAs targeting each RNA transcript, fractionated the cells, and then used qPCR to measure RNA levels. As typically observed with siRNAs, levels of all four RNA transcripts were reduced in the cytoplasmic fraction (Figure 4B). We observed a similar reduction of transcript levels in nucleoplasmic and chromatin fractions, consistent with RNAi activity in nuclei (Figure 4B).

Cleavage at a predicted location is diagnostic for substrate processing by Ago2 and we used 5′ rapid amplification of cDNA ends (5′ RACE) to further test whether silencing of RNA in the nucleoplasm and on chromatin was due to Ago2-mediated cleavage. We isolated RACE products and sequencing revealed that site-specific cleavage had occurred at the predicted phosphodiester bond in all cellular fractions for Malat1 (Figure 4C; Figure S4A-B). Identification of the predicted RACE products associated with chromatin and in nucleoplasm is additional evidence for nuclear RNAi activity.

To further investigate the potential for nuclear Ago2 to cleave RNA substrates, we set up an *in vitro* cleavage assay using Ago2 from either cytoplasmic or nuclear fractions and a radiolabeled RNA substrate derived from luciferase (Luc) mRNA (Elbashir et al., 2001). Cells were transfected with a duplex RNA (siLuc) complementary to the Luc RNA substrate or a duplex RNA containing two central mismatches (siLuc_mm). Central mismatches are known to disrupt slicer activity (Wang et al., 2008). Ago2 was then immunoprecipitated from either the cytoplasmic or nuclear fractions and incubated with the radiolabeled target RNA substrate. Reactions were then resolved on denaturing polyacrylamide gels to visualize cleavage products.

Ago2 immunoprecipitated from nuclear extracts of cells treated with siLuc caused sequence-specific cleavage of the Luc substrate (Figure 4D, lanes 6 & 9). In contrast, Ago2 from nuclear extracts of cells treated with siLuc_mm did not support cleavage (Figure 4D, lanes 7 & 10). A lack of cleavage for siLuc_mm is expected based on the known slicer mechanism of Ago2, which requires perfect complementarity at the targeted bond (Wang et al., 2008). These results demonstrate sequence-specific slicer activity for nuclear Ago2.

To visualize RNAi-mediated activity inside cell nuclei, we targeted Malat1 RNA with a siRNA (siMalat1) and performed fluorescence *in situ* hybridization (FISH). FISH prior to siMalat1 treatment revealed distinct nuclear speckles. Upon treatment with siMalat1, the Malat1 speckles disappeared. In contrast to the disappearance of speckles after treatment with siMalat1, a siRNA with no cellular target (siLuc) had no effect (Figure 4E; Figure S4C-D). Taken together, several lines of evidence are consistent with RNAi slicer activity in cell nuclei, including; 1) siRNA-mediated reduction in the levels of nuclear RNA targets, 2) site-specific cleavage of nuclear RNA targets at a position diagnostic for RNAi, 3) cleavage of target RNA by Ago 2 isolated from nuclear extract, and 4) visualization of reduced target RNA within cell nuclei.

### miRNAs in Cell Nuclei

**Figure 5:**
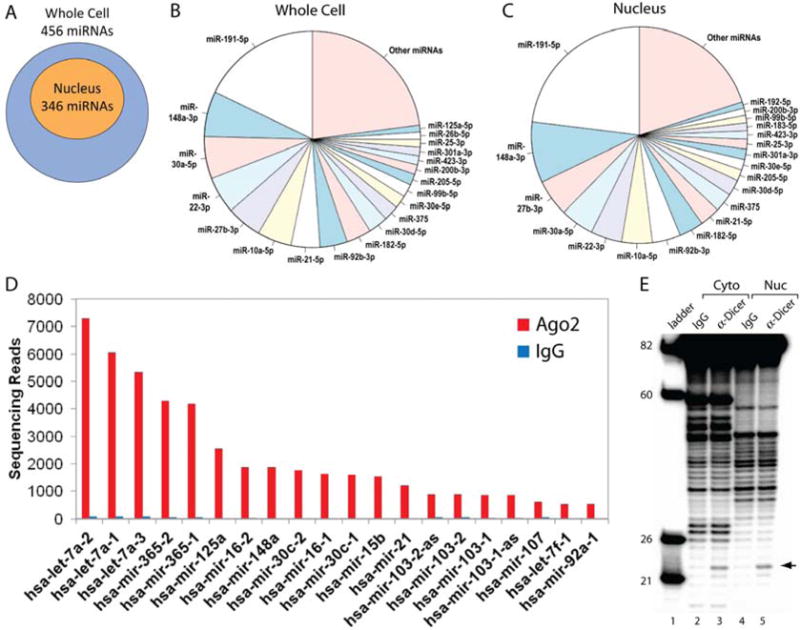
miRNAs are in the nucleus and associate with Ago2 (**A**) Distribution of miRNAs by single molecule small RNA sequencing in nuclei versus whole cell. (**B**-**C**) Relative abundance of top 20 miRNAs in whole cell versus nuclear fractions. (**D**) Top 20 miRNAs bound to nuclear Ago2 identified by immunoprecipitation and single molecule small RNA sequencing. Results are compared to a negative control from immunoprecipitation using an nonspecific antibody (IgG). (**E**) Detection of processing products from cytoplasmic and nuclear Dicer. RNA molecular weight ladder is shown to the left (lane 1).

We evaluated the localization of miRNAs within cell nuclei and their association with Ago2. Sequencing of small RNAs revealed that out of 456 miRNA species identified in the whole cell, 346 of them also exist in cell nuclei, suggesting that roughly 75% of miRNAs in the cytoplasm are shuttled into the cell nucleus (Figure 5A). The identities of many of the top 18 ranked miRNAs, based on the number of obtained sequencing reads, were the same between nuclei and whole cell (Figure 5B-C), indicating a similar distribution of abundant miRNA species in the cytoplasm and the nucleus.

We then examined the association of miRNAs with Ago2 in cell nuclei. Ago2 was immunoprecipitated from nuclear extract using a non-specific mouse IgG as a negative control. Bound small RNAs were isolated and sequenced on a Helicos single molecule sequencer using direct RNA sequencing mode. In this mode, the steps of making complementary DNA and PCR amplification are avoided so that potential sequencing biases are eliminated. The number of sequencing reads better represents the original miRNA expression level. We prepared two biological replicates and averaged the sequencing read number for each small RNA. Single molecule sequencing revealed substantial binding of numerous miRNAs to nuclear Ago2 (Figure 5C).

To further examine the potential for miRNA pathways to operate in the nucleus, we tested the ability of Dicer, the enzyme that processes pre-miRNA precursors, to generate mature miRNAs in nuclear or cytoplasmic extracts. We performed an *in vitro* processing assay in which we immunoprecipitated Dicer from cytoplasmic or nuclear extracts, mixed it with radiolabeled pre-miR-19a, and analyzed cleavage products by denaturing polyacrylamide gel electrophoresis. We observed the expected 23-nucleotide product for both nuclear- and cytoplasmic-derived Dicer (Figure 5D). These results suggest that Dicer can process pre-miRNAs in the nucleus and is consistent with the potential for nuclear regulation and production of small RNAs.

### Small Duplex RNA Loading of Ago2 is Deficient in Nuclear Extract

Observed differences in fractionation of nuclear versus cytoplasmic RNAi factor complexes suggested that other differences between cytoplasmic and nuclear RISC might exist. To extend our analysis, we tested whether there might be differences in small RNA loading between the nucleus and cytoplasm.

When examining Ago2 slicer activity *in vitro* (Figure 4D), our extracts were initially prepared from cells that were transfected with siRNA. This protocol requires the RNA to pass through the cytoplasm before entering the nucleus and therefore loading might occur in the cytoplasm. Subsequent nuclear import of loaded Ago2 complexes might then account for the slicer activity observed in the nucleus (Ohrt et al., 2008; Weinmann et al., 2009). To determine where loading was occurring, we added small RNAs directly to nuclear extracts prepared from untreated cells and subsequently performed *in vitro* assays for slicer activity.

**Figure 6:**
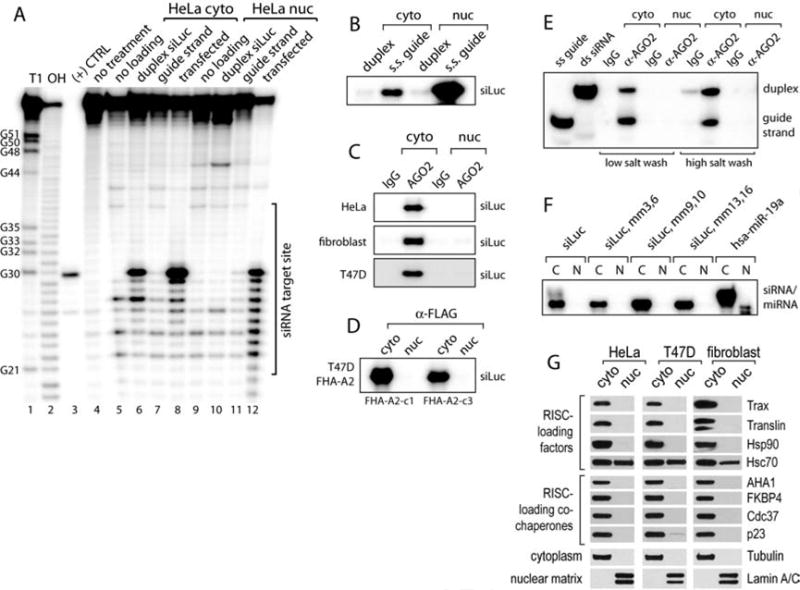
Duplex small RNA loading is deficient and RISC loading factors are absent in nuclear extracts (**A**) Cleavage of a 5′-radiolabeled luciferase RNA substrate by Ago2 isolated from cytoplasmic or nuclear fractions treated as shown above the gel. T1, RNase T1 cleavage; OH, alkaline hydrolysis; (+) CTRL, synthetic cleavage product. (**B**) Co-precipitation of radiolabeled duplex siLuc or radiolabeled single-strand siLuc guide RNA incubated with Ago2 after immunoprecipitation from nuclear or cytoplasmic extracts. (**C**-**D**) In vitro assay for Ago2 duplex siRNA loading in extracts. Radiolabeled siRNA is added to extracts from human cell lines, Ago2 immunoprecipitated with anti-Ago2 or anti-FLAG antibody, and co-purified RNA resolved on a denaturing polyacrylamide gel. FHA-A2-c1 and FHA-A2-c3 are two different T47D clonal cell lines stably expressing FLAG-HA-tagged Ago2. (**E**) Radiolabeled miR-19a or siLuc were used in the same assay shown in panels C and D. Mismatch positions relative to the 5′ end of the guide strand are indicated above the gel. (**F**) Radiolabeled siLuc was used in the same assay shown in panels C and D but co-purified RNA was resolved on a non-denaturing polyacrylamide gel. Immunoprecipitation wash conditions and shown below the gel and the co-purified duplex or single-strand RNAs indicated to the right. (**G**) Western blot of RISC loading and maturation factors and subcellular markers from cytoplasmic and nuclear fractions.

As we had observed previously, when Ago2 is isolated from extracts of cells transfected with siLuc, Ago2 from both the cytoplasm and nucleus can catalyze cleavage of the Luc substrate RNA (Figure 6A, lanes 8 and 12). By contrast, extracts from untransfected cells where double-stranded siLuc was added after extract preparation revealed that cytoplasmic Ago2 was able to catalyze cleavage but that nuclear Ago2 was inactive (Figure 6A, lanes 6 and 10). These data are consistent with nuclear slicer activity and replicate our earlier findings (Figure 2), but also suggest a deficiency in Ago2 loading in nuclear extracts.

We further examined the loading of single-stranded guide RNA alone. Single-stranded RNAs are usually rapidly degraded in cell extracts, precluding efficient loading. Nevertheless we found that addition of the single-stranded siLuc guide strand conferred similar low levels of cleavage for both cytoplasmic and nuclear Ago2 (Figure 6A, lanes 7 and 11).

RNA single-strands can be directly bound by Ago2 without the need for additional factors (Rivas et al., 2005). As a control to ensure that nuclear Ago2 was accessible for loading, we immunoprecipitated Ago2 from nuclear extract and then incubated it with a radiolabeled single-strand siLuc guide RNA. We found that both cytoplasmic and nuclear Ago2 were able to bind the single-stranded guide (Figure 6B; Figure S5A). Taken together, our observations that Ago2 from nuclear extract can; 1) direct cleavage of an RNA target after mixing with single-stranded but not duplex RNA; and 2) bind single-strand RNA, are consistent with the loading of duplex RNA being deficient in cell nuclei.

Since duplex RNAs are susceptible to degradation by nucleases (Braasch et al., 2003), we considered the possibility that nuclear extracts could harbor a nuclease activity that might interfere with loading. When duplex RNA was incubated with either nuclear or cytoplasmic extracts, we observed similar levels of degradation over time (Figure S5B), suggesting that nuclease activity cannot explain our findings.

To more directly investigate Ago2 loading with duplex small RNA, we developed an *in vitro* loading assay. We added duplex siLuc with a radiolabeled guide strand to either nuclear or cytoplasmic extracts. When Ago2 was immunoprecipitated with antibodies against either endogenous or FLAG-tagged Ago2, we observed co-purification of radiolabeled siLuc guide strand with cytoplasmic, but not nuclear, Ago2 (Figure 6C-D). A lack of nuclear loading was still observed when both siLuc strands were radiolabeled, ruling out a potential passenger strand loading bias in the nucleus (Figure S5C). We also confirmed that similar amounts of Ago2 were immunoprecipitated from both extracts in our experiments (Figure S5D). Nuclear extracts are unlikely to support efficient ATP regeneration for some RNAi processes (Klouwen and Appelman, 1967; Zamore et al., 2000). Since RISC loading has been reported to require ATP (Iwasaki et al., 2010), we used phosphocreatine and creatine kinase (Calhoun and Swartz, 2007) to regenerate ATP. We observed no change in loading in the nuclear extract (Figure S5E), excluding insufficient ATP as a possible explanation for the duplex loading deficiency.

Small duplex RNA loading involves an initial step when the duplex first binds to RISC and an unwinding step when the passenger strand is removed (Iwasaki et al., 2010; Matranga et al., 2005; Ye et al., 2011). To determine the limiting step in nuclear loading, we performed the *in vitro* Ago2 loading assay under conditions that would discriminate between duplex siRNA or single-strand guide RNA bound to Ago2. Immunoprecipitations were washed with the standard high salt (0.5 M NaCl) buffer or with a low salt (0.15 M NaCl) buffer to potentially preserve formation of unstable Ago2-RNA complexes. When RNA isolated from Ago2 immunoprecipitations was resolved by native gel electrophoresis, both duplex and single-stranded RNAs were bound to cytoplasmic Ago2, indicating formation of both complexes during loading (Fig. 6E). In contrast, neither complex was formed during loading in nuclear extracts. This result demonstrates that the initial step of duplex RNA loading is deficient in nuclear extracts.

Our observation of miRNAs and Dicer activity in cell nuclei (Figure 5) led us to examine loading of miRNAs. We tested loading of a miR-19a duplex miRNA and duplex RNAs based on siLuc that contained central or terminal mismatches. Like fully complementary RNAs, we observed that mismatched duplexes were loaded in cytoplasmic extract but were not loaded in nuclear extract (Figure 6F). These results suggest that duplex miRNAs may also be differentially loaded between the nucleus and the cytoplasm.

Since several proteins are implicated in Ago2 loading and RISC maturation (Iwasaki et al., 2010; Pare et al., 2013; Ye et al., 2011), we performed Western blot to detect their presence in isolated nuclei. Surprisingly, these loading factors, Hsp90, Translin, TRAX, Aha1, FKBP4, Cdc37 and p23, were all exclusively cytoplasmic (Figure 6G). The only exception was Hsc70, which was found in both the nucleus and the cytoplasm. These results are consistent with a loading restriction to the cytoplasm and suggest that programming of RNAi via Ago loading is regulated by exclusion of critical loading factors from the nucleus.

## Discussion

There has been conflicting evidence over the presence and activity of canonical RNAi factors in mammalian somatic cell nuclei. The biological significance of the nucleus in the regulation of RNAi pathways has also been unclear. This uncertainty has obscured the potential for small RNAs to participate in the regulation of nuclear processes. In this study we used multiple methods to test whether RNAi factors and RNAi activity could be detected in mammalian cell nuclei. We employed protocols for purifying nuclei that are free of ER protein contamination for *in vitro* analyses and methods for visualizing RNAi factors and RNAi activity in cell nuclei.

Our complementary experimental approaches support the existence of nuclear RNAi but also indicate that it differs from cytoplasmic RNAi. We find that: 1) Ago2, the catalytic engine of RNAi, and the RNAi factors Dicer, TRBP, and TNRC6A are all present in human cell nuclei; 2) these nuclear-localized RNAi factors can associate in stable multi-protein complexes; 3) small RNAs can reduce levels of nuclear-localized RNA targets through site-specific cleavage; 4) Ago2 and Dicer isolated from nuclei exhibit catalytic activity; 5) RNAi activity can be visualized inside of human cell nuclei by FISH; 6) endogenous miRNAs are bound to nuclear Ago2 and miRNA pathway components are in the nucleus; 7) programming of RNAi through Ago2 loading with duplex small RNAs is not observed in nuclear extracts; and 8) necessary RISC loading and maturation factors are absent from the nucleus.

A previous study from Meister and co-workers reported on the presence and complex formation of EGFP-tagged Ago2 in human cell nuclei using fluorescence correlation and cross-correlation spectroscopy (Ohrt et al., 2008). Consistent with their results, we also observed Ago2 in the nucleus. However, they found that nuclear EGFP-Ago2 did not appear to associate with large complexes. These differences may be accounted for by our focus on endogenous Ago2 and by variations in fractionation techniques. Based on spectroscopy data, Ohrt et al. also proposed that Ago2 is loaded in the cytoplasm and imported into the nucleus. Our data support this hypothesis with biochemical evidence and provide a potential explanation by observing a nuclear absence of the known RISC loading factors. The exclusion of RNAi programming from the nucleus has important implications for RNAi because small RNAs that are primarily nuclear may be loaded inefficiently or not at all. This partitioning of RISC loading may have evolved to regulate the involvement of nuclear small RNA in RNAi-mediated processes.

The activity and regulation of RNAi factors in mammalian cell nuclei might have multiple impacts on gene regulation. Small RNAs could potentially guide RNAi factors like Argonaute to nuclear RNA species, such as noncoding RNAs at gene promoters to affect transcription or intronic RNA to alter splicing. Nuclear RNAi-like pathways exist in various eukaryotic organisms like plants, flies, worms, fungi, and ciliated protozoa (Castel and Martienssen, 2013; Gagnon and Corey, 2012). These pathways have been characterized to regulate processes like nuclear gene expression, epigenetic states, and genome maintenance (Malone and Hannon, 2009). The demonstrated presence and activity of canonical RNAi factors in mammalian cell nuclei suggests that similar pathways may exist in humans.

We have recently reported a role for Ago2 and TNRC6A in endogenous control of the inflammatory pathway genes cyclooxygenase 2 (COX-2) and phospholipase 2G 4A (PLA2G4A) by miR-589 miRNA (Matsui et al., 2013). COX-2 and PLA2G4A are adjacent genes whose promoters are linked by chromosomal looping. miR-589 is expressed in A549 lung cancer cells and has two seed sequence target sites at the COX-2 promoter. Elevated levels of miR-589 lead to increased expression of both COX-2 and PLA2G4A. Ago2 and TNRC6A are recruited by miR-589 to a sense transcript that overlaps the COX-2 promoter. When Ago2 or TNRC6A levels are depleted, the activation of COX-2 and PLA2G4A by miR-589 is reversed. These results provide an experimental demonstration that nuclear RNAi factors can play a role in regulating a physiologically important regulatory pathway.

Mammalian RNAi has typically been assumed to localize to the cytoplasm, limiting the vision of researchers investigating the regulation of gene expression. Clarity about RNAi factors and their activity in the nucleus widens investigation of RNAi beyond traditional targets in the cytoplasm to targets in the nucleus that may regulate processes like transcription or splicing. While much remains to be learned about nuclear RNAi, such control in the nucleus would add a new layer of gene regulation and offer new options for RNAi-based therapeutics.

## Experimental Procedures

### Tissue culture and siRNA transfection

HeLa, T47D, fibroblast, and A549 cells were cultured in their standard media at 37°C in 5% CO_2_. Lipofectamine RNAiMAX (Invitrogen) was used to deliver siRNAs into cells following the manufacturer's recommended protocol. Sequences of siRNAs used are listed in Table S1.

### Nuclear and cytoplasmic cell fractions

Cells were lysed in hypotonic lysis buffer (HLB) (10 mM Tris-HCl, pH 7.5, 10 mM NaCl, 3 mM MgCl_2_, 0.3% NP-40) supplemented with 1% Protease Inhibitor (PI), 1 mM NaF, and 1 mM Na_3_VO_4_. Cells were spun and supernatant kept as cytoplasmic extract after addition of NaCl to 0.15 M and glycerol to 10%. Pelleted nuclei were washed 3× with HLB. To make nuclear extract, nuclei were resuspended in nuclear lysis buffer (same as HLB but containing 0.15 M NaCl and 10% glycerol) also supplemented with PI, NaF and Na_3_VO_4_. Nuclei were sonicated and supernatant kept as nuclear extract.

### Co-immunoprecipitation

Protein G Plus/Protein A resin (Calbiochem), antibody and pre-cleared nuclear extract were mixed at 4°C for 2-3 h. When indicated, 20 μg RNas e A was added before incubation. Resin was washed with IP wash buffer (20 mM Tris-HCl, pH 7.5, 0.4 M NaCl, 2 mM MgCl_2_, 0.05% NP-40, 0.025% SDS) and co-purified proteins eluted with SDS-PAGE loading buffer. Specific proteins were detected by Western blot.

### Chromatographic separation and ammonium sulfate cuts of cell extracts

For size-exclusion chromatography, extracts were either treated with RNase A or SUPERase-In (Ambion), filtered, injected onto a Superdex 200 HiLoad 16/60 column (Amersham Pharmacia) pre-equilibrated with FPLC buffer (20 mM Tris-HCl, pH 7.5, 150 mM NaCl, 3 mM MgCl_2_, 5% glycerol), and separated by FPLC. Eluted fractions were assayed by Western blot. For subsequent fractionation by anion exchange, size-exclusion fractions were concentrated and injected onto a Mono-Q FPLC column (Amersham Pharmacia) equilibrated with FPLC buffer at 0.1 M NaCl. Elution was performed by linear gradient from 0.1 to 1 M NaCl. For ammonium sulfate precipitation, saturated ammonium sulfate solution was added to cell extracts at the indicated final percentages, incubated on ice for 15 min, spun down at 18,000 × g at room temperature, and pelleted precipitate resuspended in SDS loading buffer. Supernatant was kept and additional ammonium sulfate added for the next cut. Fractions were analyzed by Western blot.

### Analysis of siRNA-mediated RNA knock-down in cellular compartments

HeLa cells were transfected with 25 nM siRNA then harvested 72 h later. Cells were counted and fractionated similarly to above. However, instead of sonicating, nuclei were lysed with modified Wuarin-Schibler buffer (MWS) (10 mM Tris-HCl, pH 7.0, 4 mM EDTA, 0.3 M NaCl, 1 M urea, 1% NP-40) (Wuarin and Schibler, 1994). Supernatant was kept as nucleoplasmic fraction and chromatin washed. RNA was isolated from cytoplasmic and nucleoplasmic fractions by precipitation and Trizol extraction. RNA was isolated from chromatin by Trizol extraction.

### *In vitro* Ago2 cleavage assay

HeLa cells were either untreated or transfected with 25 nM siLuc or siLuc_mm (see Table S1) then harvested 36 h later and nuclear and cytoplasmic extracts prepared. When indicated, siRNA or single-strand guide RNA was incubated with extract from untreated cells for 1 h at room temperature with rotation. Ago2 was immunoprecipitated using anti-Ago2 antibody (Abcam, ab57113), Protein G Plus/Protein A agarose (Calbiochem) and 200 μL extract at room temperature rotation for 1 h. Resin was washed with IP wash buffer (IPWB) (20 mM Tris-HCl, pH 7.5, 4 mM MgCl_2_, 0.5 M NaCl, 0.05% NP-40) then mixed with 5′ radiolabeled synthetic target RNA substrate in 1× RNAi buffer (20 mM Tris-HCl, pH 7.5, 4 mM MgCl_2_, 0.5 mM DTT, 80 mM NaCl, 20 mM KCl, 0.5 mM EDTA) supplemented with 1 mg/mL yeast tRNA, 20 units SUPERase-In (Ambion), and 0.5 mM ATP. Reactions were incubated at 30°C for 1.5 hr with periodic mixing then target RNA and cleavage products phenol extracted. Extracted RNA was resolved on a 15% denaturing polyacrylamide sequencing gel. The gel was dried and exposed to a phosphorimager screen overnight to visualize radioactive bands.

### Immuno-fluorescence (IF) and co-localization analysis

Immuno-fluorescence was performed similarly to that previously described (Ohrt et al., 2012; Spector, 2011) with modifications. Briefly, cells were grown on 35 mm dishes with a 14 mm glass bottom. Cells were fixed in 2% formaldehyde or 4% paraformaldehyde. Fixed cells were then permeabilized with 0.2% Triton X-100 or 70% ethanol. Cells were incubated in primary antibody in PBS + 1% normal goat serum (NGS), washed, incubated with secondary antibody + 1% NGS, washed again, then set in mounting medium with DAPI and imaged. For immuno-fluorescence of isolated nuclei, fixing and antibody incubations were all performed in suspension before spotting to slides for imaging. Cells were imaged by wide-field epifluorescence microscopy and images processed by blind deconvolution with AutoQuant X3 (Media Cybernetics). Alternatively, some samples were imaged by Andor spin disc confocal microscopy. Co-localization channels were calculated using Imaris (Bitplane) based on the correlation of the strength of linear relation between the two channels. Threshold levels for calculation were selected above background.

### Fluorescence *in situ* hybridization (FISH)

Cells were grown on 35mm MatTek dishes and transfected with 25 nM siLuc or siMalat1 as described above. Cells were fixed in ice-cold 4% PFA and permeabilized in 70%. From this point forward, the protocol recommended by the manufacturer of the FISH probes for Malat-1 (Biosearch Technologies, New Stellaris RNA FISH Probe for Malat-1, SMF-2035-1) was followed. Cells were set with mounting medium with DAPI and imaged as above for IF.

### Small RNA sequencing

Small RNA-seq libraries were constructed from either whole cell RNAs or nuclear RNAs isolated from T47D cells and sequenced on Illumina Hiseq 2000. The reads were aligned to human genome hg19, UCSC miRNA database, and/or miRBase (mature miRNA). Ago2-associated miRNA in cell nucleus was identified by first carrying out a RNA immunoprecipitation using a specific anti-Ago2 antibody. Small RNA (<40nt) including miRNA was further gel-selected. The small RNA was then subjected to poly-A tailing and sequenced on a single-molecule Helicos sequencer with a Direct RNA Sequencing (DRS) module.

### *In vitro* Ago2 small RNA loading assay

Duplex siRNA or single-strand guide RNA radiolabeled at the 5′ end was incubated with extract supplemented with 1 mM ATP for 1 h at room temperature with rotation. Ago2 was immunoprecipitated using anti-Ago2 antibody (Abcam, ab57113) and Protein G Plus/Protein A agarose (Calbiochem). Resin was washed with IP wash buffer (IPWB) (20 mM Tris-HCl, pH 7.5, 4 mM MgCl_2_, 0.5 M NaCl, 0.05% NP-40) then phenol-chloroform extract to isolate co-purified RNA. Extracted RNA was resolved on a 15% denaturing polyacrylamide sequencing gel or a 15% native TBE-buffered polyacrylamide gel. The gel was dried and exposed to a phosphorimager screen overnight to visualize radioactive bands.

## Supplementary Material

01

02

03

## Highlights

- RNAi factors associate in multi-protein complexes in the human cell nucleus.
- Execution of RNAi, but not RNA loading of Argonaute, occurs in the nucleus.
- RNAi complexes and activities differ between the cytoplasm and nucleus.
