# Supplementary material for: RNAi Factors are Present and Active in Human Cell Nuclei
Source: Cell Rep. Author manuscript; Available in PMC 2015 Jan 16. (PMC3916906; doi:10.1016/j.celrep.2013.12.013)
Supplement: 02 [file NIHMS550334-supplement-02.pdf]

RNAi Factors are Present and Active in Human Cell Nuclei

Keith T. Gagnon*, Liande Li*, Yongjun Chu, Bethany A. Janowski, and David R. Corey

Departments of Pharmacology and Biochemistry, University of Texas Southwestern Medical Center, Dallas, Texas, 75390-9041.

*These authors contributed equally.

SUPPLEMENTAL INFORMATION

SUPPLEMENTAL EXPERIMENTAL PROCEDURES

Tissue culture and siRNA transfection

HeLa cells were cultured in Dulbecco's Modified Eagle's Medium (DMEM) supplemented with 5% fetal bovine serum (FBS) and 0.5% non-essential amino acids (NEAA). T47D cells (ATCC) were cultured in RPMI medium supplemented with 10% FBS, 0.5% NEAA, 20 μg/mL insulin, 10 mM pH 7.0-7.6 HEPES, and 1 mM sodium pyruvate. Fibroblast cells (Coriell Institute, GM04281) were cultured in minimum essential medium (MEM) supplemented with 10% FBS and 0.5% NEAA. A549 cells (ATCC) were cultured in F-12K medium supplemented with 10% FBS. T47D cells stably expressing FLAG-HA-tagged Ago2 were cultured identically to T47D cells but media supplemented with 0.2 mg/mL G418. All cells were grown at 37°C in 5% CO_2.

Lipofectamine RNAiMAX (Invitrogen) was used to deliver siRNAs into HeLa cells following the manufacturer's recommended protocol in OptiMEM low serum medium (Invitrogen). Growth media was changed to full medium after 24 h. Transfected cells were harvested 72 h after transfection for qPCR and RACE analyses, 36 h after transfection for in vitro Ago2 cleavage assays, and 48 h after transfection for FISH analysis. Sequences of siRNAs used are listed in Table S1.

Nuclear and cytoplasmic cell fractions

Cells were harvested with trypsin-EDTA solution (Invitrogen), washed with PBS then resuspended in ice-cold hypotonic lysis buffer (HLB) (10 mM Tris-HCl, pH 7.5, 10 mM NaCl, 3 mM $MgCl_2$, 0.3% NP-40) supplemented with 1% Protease Inhibitor Cocktail Set I (Calbiochem), 1 mM sodium fluoride and 1 mM sodium orthovanadate at a final of 1 mL/75 mg wet cell pellet. After incubation on ice for 15 min and pipetting and vortexing, lysate was

spun at 4°C at 800xg for 5 min. The supernatant was kept as cytoplasmic extract and NaCl and glycerol were added to a final of 140 mM and 10%, respectively. Pelleted nuclei were washed 3x with ice-cold HLB by 5 min incubation on ice, pipetting and vortexing, then spinning at 4°C at 100xg for 2 min. For nuclear extracts, nuclei were resuspended in ice-cold nuclear lysis buffer (NLB) (20 mM Tris-HCl, pH 7.5, 0.15 M NaCl, 3 mM $MgCl_2$, 0.3% NP-40, 10% glycerol) supplemented with 1% Protease Inhibitor Cocktail Set I, 1 mM sodium fluoride and 1 mM sodium orthovanadate at a final of 0.5 mL/75 mg of original wet cell pellet weight (1/2 the volume of cytoplasmic fraction). Nuclei were sonicated on ice at 20% power 3x for 15 sec in 4 mL volumes. After high speed centrifugation at 4°C to remove insoluble cell debris, the soluble fraction was kept as nuclear extract. All extracts were aliquoted, flash-frozen in liquid nitrogen, then stored at -80°C for later use.

Optimization of nuclei isolation protocol

A protocol similar to the above described for nuclear and cytoplasmic fraction preparation was followed except detergent was either omitted or TWEEN-20, NP-40 or Triton X-100 non-ionic detergents were included in HLB at the indicated concentrations. After the final nuclei wash, a fraction of the nuclei were aliquoted for visualization by fluorescence microscopy while the remaining nuclei were resuspended in NLB and sonicated to prepare nuclear extracts as described above. Nuclei were prepared for microscopy by washing 1x in ice-cold PBS then incubating in PBS + 1 µM ER Tracker Red (Invitrogen) for 20 min on ice. Nuclei were diluted 10-fold in ice-cold PBS + 4% paraformaldahyde and incubated on ice 10 min. Nuclei were resuspended by pipetting and spotted on glass slides. After partial air-drying, one drop of Vectashield Hard Set Mounting Medium with DAPI (Vector Laboratories, H-1500)

was added, a coverslip added and mounting media allowed to harden at room temperature for 15 min.

Nuclei were visualized with a 60x objective lens and DAPI and TRITC filters on a wide-field epifluorescence Deltavision microscope. Z-sections were taken at 0.15 μm thickness. Images were deconvoluted by blind deconvolution using AutoQuant X3 (Media Cybernetics), stacked and ER tracker staining pseudo-colored yellow in ImageJ for visualization.

Western blot analysis

Cell extracts were prepared as described above. For comparing nuclear and cytoplasmic fractions by Western blot, the same cell equivalents of extract were separated by electrophoresis (1/2 the volume of nuclear extract for every 1 volume of cytoplasmic). In general, loading equal amounts of total protein is unsatisfactory for comparing the nuclear and cytoplasmic levels of specific proteins since there is approximately 4-fold more total protein in cytoplasmic extracts. Protein was separated on 4-20% gradient SDS-PAGE TGX pre-cast gels (Biorad) at 100 V for 75 min. After gel electrophoresis, proteins were transferred to nitrocellulose membrane (Hybond-C Extra, GE Healthcare Life Sciences) at 100 V for 90 min. Membranes were blocked for 30 min at room temperature with 5% milk protein in PBS + 0.05% TWEEN-20 (PBST).

Blocked membranes were incubated with the following specific primary antibodies at 4°C in PBST + 5% milk with rocking overnight: anti-Ago2 at 1:1000 (Abcam, ab57113), anti-TNRC6A at 1:4000 (Bethyl Laboratories, A302-329A) or anti-TNRC6A at 1:3000 (kind gift from Edward Chan, Univ. Florida, rb5182), anti-Dicer at 1:1000 (Abcam, ab14601), anti-TRBP at 1:1000 (Abcam, ab72110) or anti-TRBP at 1:3000 (kind gift from Qinghua Liu lab); anti-Calreticulin at 1:1500 (Cell Signaling, 2891S), anti-Calnexin at 1:1500 (Cell Signaling,

2433S), anti-Histone H3 at 1:10000 (Abcam, ab1791), anti-Lamin A/C at 1:1500 (Abcam, ab8984), anti-OxPhos at 1:1000 (Invitrogen, A21351), anti-tubulin at 1:6000 (Sigma-Aldrich, T5201), anti-Ago1 at 1:1000 (Wako Chemical, 015-22411), anti-Ago3 at 1:500 (Active Motif, 39788); anti-Ago4 at 1:500 (Active Motif, 39856), anti-PACT at 1:1000 (Abcam, ab75749), anti-Hsp90 at 1:500 (Enzo Life Sci., ADI-SPA-830-D), anti-Hsc70 at 1:500 (Enzo Life Sci., ADI-SPA-810-D), anti-Aha1 at 1:1000 (Abcam, ab56721), anti-FKBP4 at 1:50000 (Abcam, ab124906), anti-Cdc37 at 1:50000 (Abcam, ab108305), anti-p23 at 1:1000 (Abcam, ab2814).

After primary antibody incubation, membranes were washed 3x for 5 min at room temperature with PBST then incubated for 30-45 min at room temperature with HRP-conjugated anti-mouse at 1:10000 (Jackson Laboratories, 715-035-150) or anti-rabbit at 1:5000 (Jackson Laboratories, 711-035-152) in PBST + 5% milk. Membranes were washed again 3x for 15 min in PBST at room temperature, then protein bands visualized using SuperSignal West Pico Chemiluminescent Substrate (Thermo Scientific). For quantification of RNAi factor protein levels from Western blots of cellular fractions, films were scanned and bands quantified using ImageJ.

Co-immunoprecipitation

Co-immunoprecipitation experiments were performed by mixing 40 µl Protein G Plus/Protein A resin (Calbiochem), 2 µg antibody and nuclear extract (~0.5-1 mg total protein, precleared at 4°C for 30 min using ~15 µL Protein G Plus/Protein A resin and ~0.3 µg of corresponding IgG) and rotating at 4°C for 2-3 h. Resin was washed 4x with IP wash buffer (20 mM Tris-HCl, pH 7.5, 0.4 M NaCl, 2 mM $MgCl_2$, 0.05% NP-40, 0.025% SDS) and co-purified proteins eluted by boiling resin in 25 µl 1x SDS loading buffer. Eluted protein was resolved by SDS-PAGE and proteins detected by Western blot as described above. When indicated, 20 µg

RNase A was added before incubation. For FLAG-tagged Ago2 co-immunoprecipitation, about 20 µL ANTI-FLAG-M2 affinity gel (Sigma) and nuclear extract from T47D cells stably expressing FLAG-HA-tagged Ago2 (~0.5-1 mg total protein) were rotated at 4°C for ~1.5-2.5 h, followed by washing and elution as described above.

Chromatographic and ammonium sulfate fractionation of cell extracts

Nuclear extracts were prepared as described above but concentrated 2-fold by using 1/2 the standard amount of NLB during nuclei resuspension and sonication. For size-exclusion chromatography, 2 mL of nuclear extract was either treated with 50 µg of RNase A or 200 units of SUPERase-In (Ambion) for 1 h at room temperature. Samples were then 0.45 µm filtered and injected onto a Superdex 200 HiLoad 16/60 FPLC column (Amersham Pharmacia) that was pre-equilibrated with FPLC buffer (20 mM Tris-HCl, pH 7.5, 150 mM NaCl, 3 mM $MgCl_2$, 5% glycerol). Protein was eluted off the column with FPLC buffer and the elution collected in fractions at 4°C that were then snap-frozen in liquid nitrogen and stored at -80°C. Western blot analysis of fractions was performed as described above. For subsequent fractionation by anion exchange, size-exclusion fractions were concentrated 3-fold and diluted to 0.1 M NaCl and injected onto a Mono-Q FPLC column (Amersham Pharmacia) equilibrated with FPLC buffer at 0.1 M NaCl. Elution was performed at room temperature with a linear gradient from 0.1 to 1 M NaCl in FPLC buffer. Fractions were collected and snap-frozen in liquid nitrogen. Western blot analysis was performed as described above.

Ammonium sulfate cuts were performed by addition of saturated ammonium sulfate solution to extract up to the indicated percentage and incubated on ice for 15 min. Precipitated protein was pelleted by centrifugation at 18,000xg for 20 min at 4°C. Supernatant was kept and additional ammonium sulfate added up to the next indicated percentage and

incubation and centrifugation repeated. Precipitated protein pellets were resuspended in identical volumes of SDS loading buffer, resolved by SDS-PAGE and probed by Western blot as described above.

Analysis of siRNA-mediated RNA knock-down in cellular compartments

HeLa cells were transfected in quadruplicate with 25 nM siRNA then harvested 72 h later with trypsin-EDTA solution. Cells were washed with PBS then counted with a hemocytometer. Five million cells were aliquoted and resuspended in 380 µL of ice-cold hypotonic lysis bufer (HLB) (10 mM Tris, pH 7.5, 10 mM NaCl, 3 mM $MgCl_2$, 0.3% NP-40) and incubated on ice for 15 min. Lysate was pipetted and vortexed then spun at 500xg at 4°C for 5 min. Supernatant was kept as cytoplasmic fraction, 40 µL of 3 M sodium acetate, pH 5.5, added, 100% ethanol added up to 70%, and precipitated at -20°C overnight.

Pelleted nuclei were washed 3x with ice-cold HLB and pipetting and vortexing then resuspended in 380 µL of ice-cold modified Wuarin-Schibler buffer (MWS) (10 mM Tris-HCl, pH 7.0, 4 mM EDTA, 0.3 M NaCl, 1 M urea, 1% NP-40) (Wuarin and Schibler, 1994). Sample was incubated on ice for 30 min. with vortexing every 10 min., then spun at 500xg at 4°C for 5 min. Supernatant was kept as nucleoplasmic fraction, 40 µL of 3 M sodium acetate, pH 5.5, added, 100% ethanol added up to 70%, and precipitated at -20°C overnight.

Pelleted chromatin was washed 3x with ice-cold MWS then 1 mL Trizol (Invitrogen) was added to the final chromatin pellet. To process cytoplasmic and nucleoplasmic fractions, precipitates were pelleted at 18000xg for 15 min at 4°C then 1 mL Trizol added to each pellet. Samples in Trizol were heated to 70°C with vortexing until completely dissolved, then cooled to room temperature. To each sample 0.2 mL chloroform:isoamyl alcohol (24:1) was added, samples vortexed, then spun at 18000xg for 10 min. The top aqueous layer was collected

and RNA precipitated by addition of 1 volume of isopropanol and incubation at -20°C overnight. RNA was pelleted by spinning at 18000xg, washed with 70% ethanol, then air dried and prepared for quantitative PCR or 5' RACE.

Quantitative PCR

Identical volumes of RNA (representing approximately the same number of cells and ranging from 1-2 µg of RNA) that were prepared from cellular fractions above were treated with 2 units of DNase I (Worthington) in 9.5 µL of DNase I buffer (10 mM Tris-HCl, pH 7.0, 10 mM NaCl, 2 mM $MgCl_2$, 0.5 mM $CaCl_2$) for 15 min at room temperature to degrade any genomic DNA contamination. Afterwards, 0.5 µL of a 50 mM EDTA, 10 mM EGTA solution was added and DNase I heat-inactivated at 70°C for 10 min. Treated RNAs were reverse-transcribed using the High Capacity cDNA Reverse Transcription Kit (Applied Biosystems) in a final volume of 20 µL. Quantitative PCR (qPCR) was performed using iTaq Supermix with ROX (Biorad) with ~10-20 ng of cDNA as template.

Data were normalized relative to measured GAPDH levels in each cellular compartment. Because no normalization control exists that is the same level across cellular compartments, and because spike-in controls can have variability in performance (data not shown), comparisons among treatments were only performed within each cellular compartment and not across cellular compartments (ie. chromatin to chromatin, not cytoplasm to chromatin). Primers used in qPCR are listed in Table S1.

Rapid Amplification of cDNA Ends (RACE)

RACE was performed using the GeneRacer Kit (Invitrogen) following the manufacturer's recommended protocol. cDNA was prepared from ~1 µg RNA from each cellular fraction

(prepared as described above) by reverse-transcription (RT) reaction using random primers. The 5’ end of cDNA was amplified using Platinum Taq DNA polymerase (Invitrogen) and specific primer sets for Malat1 and RPL30 (Table S1). The thermal cycling condition was 94°C for 2 min, followed by 5 cycles of 94°C for 30 sec and 72°C for 1 min, 5 cycles of 94°C for 30 sed and 70°C for 1 min, and 25 cycles of 94°C for 30 sec, 65-66°C for 30 sec, and 68°C for 1 min, followed by final extension of 68°C for 10 min. PCR products were analyzed on 1.2-1.5% agarose gel (Fig. S6B-C). Major PCR products on gels were excised and cloned intopCR 4-TOPO vectors, transformed into TOP10 chemically competent cells, then sequenced to map the 5’ cleavage sites. Chromatin-associated cleavage products for RPL30 were below our detection limit (Fig. S6C), despite performing nested PCR with multiple primer sets (data not shown). This result may reflect the relatively low level of RPL30 mRNA actually associated with chromatin. In addition to relative starting levels of targeted RNA, detection of cleavage products will also depend upon other factors like the rate of cleavage product formation and degradation.

In vitro Ago2 cleavage assay

HeLa cells were either untreated or transfected with 25 nM siLuc or siLuc_mm (see Table S1) then harvested 36 h later and nuclear and cytoplasmic extracts prepared as described above. Ago2 protein was immunoprecipitated using 1.5 µg anti-Ago2 antibody (Abcam, ab57113), 30 µL Protein G Plus/Protein A agarose (Calbiochem) and 200 µL extract with rocking at room temperature for 1 h. Resin was washed 3x with 0.5 mL IPWB$_{500}$ (20 mM Tris-HCl, pH 7.5, 4 mM $MgCl_2$, 0.5 M NaCl, 0.05% NP-40) then 1x with 0.5 mL IPDB (20 mM Tris-HCl, pH 7.5, 3 mM $MgCl_2$, 0.15 M NaCl) at room temperature. Synthesized target RNA substrate was 5' radiolabeled with T4 polynucleotide kinase and [[illegible]]^{32}P-ATP, then gel-purified. Labeled target

RNA substrate (50,000 cpms, ~0.1 pmols) was added to the washed Ago2-bound resin in 20 µL 1X RNAi buffer (20 mM Tris-HCl, pH 7.5, 4 mM $MgCl_2$, 0.5 mM DTT, 80 mM NaCl, 20 mM KCl, 0.5 mM EDTA) supplemented with 1 mg/mL yeast tRNA, 20 units SUPERase-In (Ambion), and 0.5 mM ATP.

Reactions were incubated at 30°C for 1.5 hr with periodic mixing and RNA collected by addition of 0.5 µL of 0.5 M EDTA then phenol extracted. Extracted RNA was precipitated with 9 volumes of 2% $LiClO_4$ in acetone, washed with acetone, resuspended in 90% formamide, 1x Tris-Borate EDTA (TBE) buffer, boiled for 3 min, then resolved on a 15% denaturing polyacrylamide (7 M urea, 1x TBE, 2% glycerol) sequencing gel. The gel was dried and exposed to a phosphorimager screen overnight to visualize radioactive bands. Target RNA substrate consists of a single siLuc siRNA target site flanked by firefly luciferase gene sequence and capped with two terminal DNA bases on each end. Target RNA substrate sequence (DNA bases capitalized, complementary siRNA target site in brackets):
GAacaauugauuuuacagac[gcacauaucgaggugaaca]ucacguacgcggaauacuTC.
Synthetic product RNA sequence: GAacaauugauuuuacagac[gcacauauc.

Immuno-fluorescence and co-localization analysis

Immuno-fluorescence was performed similarly to that previously described(Ohrt et al., 2012; Spector, 2011) with modifications. Briefly, cells were grown for 16-24 h to 50-70% confluency on 35 mm dishes (MatTek Corporation, P35GCOL-1.5-14-C) with a 14 mm glass bottom of 1.5 mm thickness. Cells were washed with PBS then fixed in freshly made 2% formaldehyde in PBS or 4% paraformaldehyde in PBS for 15 min at 20°C, or in 70% ethanol for at least 30 min at 4°C. Fixed cells were washed 3x in PBS for 10 min at 4°C. Cells were permeabilized in

PBS containing 0.2% Triton X-100 and 1% normal goat serum (NGS) for 5-10 min on ice then washed 3x with ice-cold PBS + 1% NGS at 4°C for 10 min.

The cells were incubated in primary antibody for 1 hr at room temperature or overnight at 4°C. Primary antibodies were diluted in PBS + 1% NGS and incubated for 1 h at room temperature or at 4°C overnight: anti-Ago2 at 1:100 (Abcam, ab57113), anti-Ago2 at 1:50 or 1:100 (Sigma-Aldrich, clone 11A9, SAB4200085), anti-Ago2 at 1:50 or 1:100 (Wako Chemicals, 015-22031), anti-TNRC6A at 1:50 or 1:100 (Bethyl Laboratories, A302-329A), anti-Dicer at 1:100 (Abcam, ab14601), anti-TRBP at 1:100 (Abcam, ab72110), or anti-PACT at 1:100 (Abcam, ab75749). Cells were washed 3x in PBS + 1% NGS for 10 min at room temperature, then incubated in 4 μg/mL secondary antibody diluted in PBS + 1% NGS for 1 h at room temperature. Secondary antibodies were Alexa Fluor 488 Goat Anti-Mouse IgG (Invitrogen, A-11001), Alexa Fluor 594 Goat Anti-Rabbit IgG (Invitrogen, A-11012), and Alexa 594 Goat Anti-Rat IgG (Invitrogen, A-11007). Cells were washed 4x in PBS for 10 min at room temperature then Vectashield Hard Set Mounting Medium with DAPI (Vector Laboratories, H-1500) was added to the cells then covered with a coverslip and allowed to harden for 15 min at room temperature.

Cells were imaged with a 60x objective lens and DAPI, FITC and TRITC filters on a wide-field epifluorescence Deltavision deconvolution microscope. Z-sections were taken at 0.1, 0.15 or 0.2 μm for at least 6 μm thickness. Images were blind deconvoluted using AutoQuant X3 (Media Cybernetics) and stacked and analyzed in Imaris (Bitplane). Alternatively, some samples were imaged by Andor spin disc confocal microscopy. The Co-localization channel of Ago2 (FITC, green) and TNRC6A (TRITC, red) was calculated using Imaris software based on the correlation of the strength of linear relation between the red

channel and the green channel and the threshold levels for calculation of co-localization were selected above background signals.

Fluorescence *in situ* hybridization (FISH)

Cells were grown for 16-24hrs to 50-70% confluence on 35mm MatTek dishes as described for immuno-fluorescence above. Cells were transfected with 25 nM siLuc or siMalat1 as described above. At 48 h post transfection, cells were washed with PBS and fixed in ice-cold 4% paraformaldehyde in PBS for 15 min at 20°C, followed by permeabilization in 70% ethanol for at least 30 min at 4°C.

From this point forward, the protocol recommended by the manufacturer of the FISH probes for Malat-1 (Biosearch Technologies, New Stellaris RNA FISH Probe for Malat-1, SMF-2035-1) was followed with modifications. Briefly, cells were washed with 1 mL of wash buffer (10% formamide in 2x SSC) and incubated 5 min at room temperature, then incubated in hybridization buffer (10% formamide, 2x SSC, 100 mg/mL dextran sulfate, 1 mM vanadyl ribonucleoside complex) containing Malat-1 probe (125 nM) in a humidified chamber at 37°C for 4-16 h in the dark. Cells were washed 1x with wash buffer in the dark at 37°C for 30 min. Vectashield Hard Set Mounting Medium with DAPI (Vector Laboratories, H-1500) was then added, covered with a coverslip and allowed to harden for 15 min at room temperature. Cells were imaged and analyzed the same as described above for immuno-fluorescence but using DAPI and Cy-5 filters. Z-stacks were taken with 0.15 or 0.2 μm slices and 6 μm thickness.

Small RNA-seq library preparation and sequencing

For whole cell small RNA sequencing, T47D cells were harvested and then dissolved in TRIzol (Sigma). For sequencing of small RNA from cell nuclei, pure nuclei were isolated first

as described above. Nuclei were then dissolved in TRIzol. Small RNA was isolated using miRNeasy Mini Kit (Qiagen) and then treated with DNase I (Worthington) for 20 min at 37°C to remove any contaminating genomic DNA. The small RNA-seq library was made using Illumina Small RNA Truseq kit following the manufacturer's recommended protocol. RNA-seq libraries were sequenced on a Illumina HiSeq 2000 as per manufacturer's instructions for single-end 1x50. Approximately, 30 million raw reads (averaged over two replicates) were obtained per sample. Reads with low quality score were removed and reads that passed the filtration were trimmed by removing the adaptor sequence. Trimmed reads shorter than 15 nt were also excluded from analysis. Filtered and trimmed reads were aligned to the human genome (hg19) using Bowtie2 by allowing up to two mismatches to the reference sequence. Up to 10 different alignments per read were permitted. For reads that were aligned to multiple positions in the reference genome, the single aligned read with the fewest mismatches was selected using a Perl script. If multiple reads still remained, the original read would be disregarded. Approximately 70% of raw reads were successfully aligned. Finally, the aligned reads were again mapped to the UCSC miRNA database and miRBase (mature miRNAs) to search for possible miRNA hits.

Sequencing of AGO2-associated small RNA in cell nuclei

RNA Immunoprecipitation (RIP) assay using nuclear lysate (T47D cells) was performed as described (Chu et al., 2010; Matsui et al., 2013) using anti-human Ago2 antibody (Wako Chemical, 015-22031). A non-specific mouse IgG antibody was used as a control. The isolated RNA was loaded on a 10% polyacrylamide gel and the small RNA fraction (15nt-40nt) was cut out and extracted. The small RNA was then subjected to polyadenylation by using a poly(A) tailing kit (Ambion). 3'-deoxy-ATP (cordycepin triphosphate, Jena

Biosciences) was introduced 10 min after the initiation of the polyadenylation reaction for 3'-end blocking and tail length limitation (performed by Helicos, Inc.). The Direct RNA Sequencing (DRS) was carried out on a single molecule Helicos sequencer. Raw sequencing data was filtered to remove low quality reads and subsequently aligned using the Helisphere package, a software designed to specifically analyze sequencing data generated from the Helicos sequencer. The aligned reads were again mapped to the UCSC miRNA database and miRBase (mature miRNAs) to search for possible miRNA hits.

***In vitro* Dicer processing assay**

Dicer was immunoprecipitated using 3 μg of anti-Dicer (Abcam, ab14601) antibody (or 3 μg normal mouse IgG as control), 50 μL Protein G Plus/Protein A agarose (Calbiochem) and 200 μL of HeLa nuclear or cytoplasmic extract (prepared as described above) with rotation at room temperature for 1 h. Resin was washed 3x with 0.5 mL $IPWB_{300}$ (20 mM Tris-HCl, pH 7.5, 4 mM $MgCl_2$, 0.3 M NaCl, 0.05% NP-40) then 1x with 0.5 mL IPDB (20 mM Tris-HCl, pH 7.5, 3 mM $MgCl_2$, 0.15 M NaCl) at room temperature. Pre-miR-19a substrate was *in vitro* transcribed in the presence of $[\alpha]^{32}$P-ATP then gel-purified. Radiolabeled pre-miR-19a RNA (50,000 cpms) was added to the washed Dicer-bound resin in 20 μL 1X Dicer assay buffer (40 mM Tris-HCl, pH 7, 3 mM $MgCl_2$, 50 mM NaCl) supplemented with 2 mM ATP and 200 units SUPERase-In (Ambion).

Reactions were incubated at 32°C for 1 hr with periodic mixing and RNA collected by addition of 0.5 μL of 0.5 M EDTA and 5 μg yeast tRNA then phenol extracted. Extracted RNA was precipitated with 9 volumes of 2% $LiClO_4$ in acetone, washed with acetone, resuspended in 90% formamide, 1x Tris-Borate EDTA (TBE) buffer, boiled for 3 min, then resolved on a 15% denaturing polyacrylamide (7 M urea, 1x TBE, 2% glycerol) sequencing gel. The gel was

dried and exposed to a phosphorimager screen overnight to visualize radioactive bands. Pre-miR-19a sequence from miRBase was used prepare *in vitro* transcription template. DNA seqeunces below were annealed and used for *in vitro* transcription (Epicentre Ampliscribe T7 In Vitro Transcription Kit) following the manufacturers protocol.

hsa-miR-19a pre-miRNA T7 sense (lowercase = T7 promoter):

ctaatacgactcactataGCAGTCCTCTGTTAGTTTTGCATAGTTGCACTACAAGAAGAATGTAGT TGTGCAAATCTATGCAAAACTGATGGTGGCCTGC

hsa-miR-19a pre-miRNA T7 antisense (lowercase = T7 promoter):

GCAGGCCACCATCAGTTTTGCATAGATTTGCACAACTACATTCTTCTTGTAGTGCAACTA TGCAAAACTAACAGAGGACTGCtatagtgagtcgtattag

***In vitro* Ago2 small RNA loading assay**

Duplex siRNA or single-strand guide RNA radiolabeled at the 5' end was incubated with cell extracts supplemented with 1 mM ATP for 1 h at room temperature with rotation. For reactions using mismatch-containing siLuc, the standard antisense siLuc strand was radiolabeled and annealed to sense strand with the indicated base position mismatches (see Table S1) then gel-purified before use. When indicated, phosphocreatine (10 mM) and creatine kinase (100 μg/mL) were added as an ATP regeneration system. Ago2 was immunoprecipitated using 2μg of anti-Ago2 antibody (Abcam, ab57113) and 40 μL of Protein G Plus/Protein A agarose (Calbiochem) with rotation for 1 h at room temperature. Resin was washed 3x with 0.8 mL $IPWB_{500}$ (20 mM Tris-HCl, pH 7.5, 4 mM $MgCl_2$, 0.5 M NaCl, 0.05% NP-40) and 1x with 0.5 mL IPDB (20 mM Tris-HCl, pH 7.5, 3 mM $MgCl_2$, 0.15 M NaCl) at room temperature. Co-precipitated RNA was collected by addition of 0.5 μL of 0.5 M EDTA and 5 μg yeast tRNA then phenol-chloroform extraction. Extracted RNA was precipitated with

9 volumes of 2% $LiClO_4$ in acetone, washed with acetone, resuspended in 90% formamide, 1x Tris-Borate EDTA (TBE) buffer, boiled for 3 min, then resolved on a 15% denaturing polyacrylamide (7M urea, 1x TBE, 2% glycerol) sequencing gel. The gel was dried and exposed to a phosphorimager screen overnight to visualize radioactive bands.

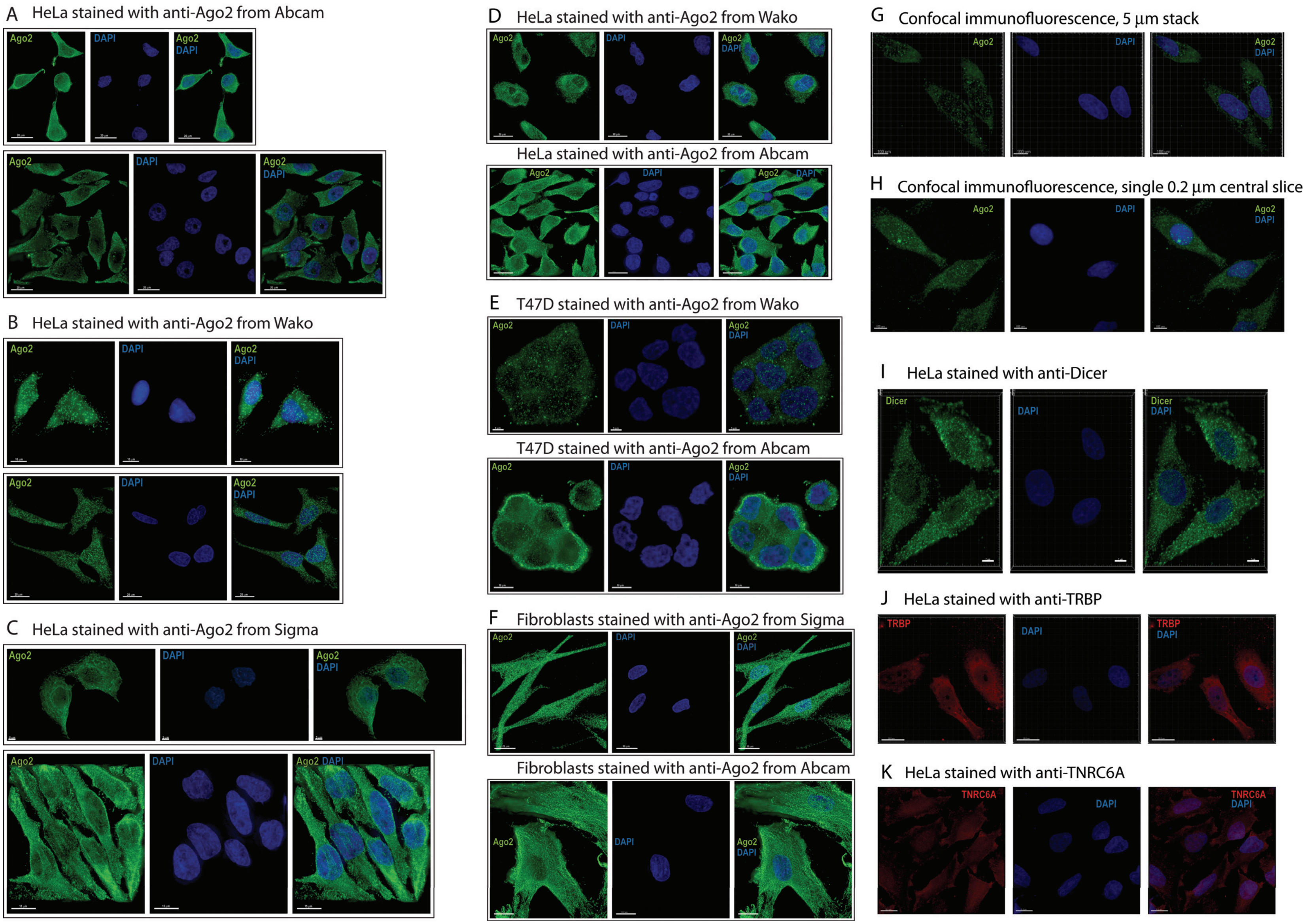

<u>Figure S1, Related to Figure 1</u>: Staining of Ago2 and other RNAi factors is observed in the nucleus of multiple human cell lines with multiple antibodies. Cells were fixed, permeabilized and immuno-stained identically except for the use of different primary and secondary antibodies (see Methods). (**A-C**) HeLa stained with anti-Ago2 from Abcam, Wako or Sigma-Aldrich and imaged by wide-field epi-immunofluorescence microscopy with blind deconvolution. Z-stacks are 5 μM thick for all images. (**D-F**) HeLa, T47D or fibroblast cells stained with anti-Ago2 from Abcam, Wako, or Sigma-Aldrich and imaged by wide-field epi-immunofluorescence microscopy with blind deconvolution. Z-stacks are 5 μM thick for all images. (**G-H**) HeLa cells stained with anti-Ago2 from Abcam and imaged by spinning disc confocal immunofluorescence microscopy. (**I-K**) HeLa cells stained with anti-Dicer, anti-TRNC6A, or anti-TRBP and imaged by wide-field epi-immunofluorescence microscopy with blind deconvolution. Z-stacks are 6 μm thick (panel I), 4 μm thick (panel J), and 6 μm thick (panel K).

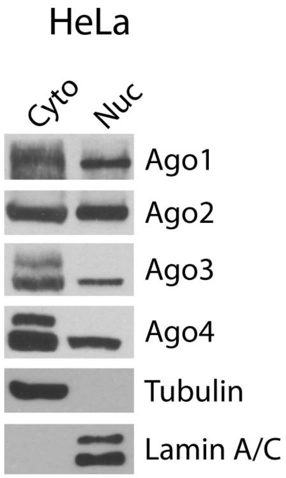

Figure S2, Related to Figure 2: Argonaute family proteins are detected in HeLa nuclear extracts by Western blot. Cell extracts were prepared as described and probed by Western blot with specific antibodies to detect each Ago family protein, Ago1, Ago2, Ago3 and Ago4.

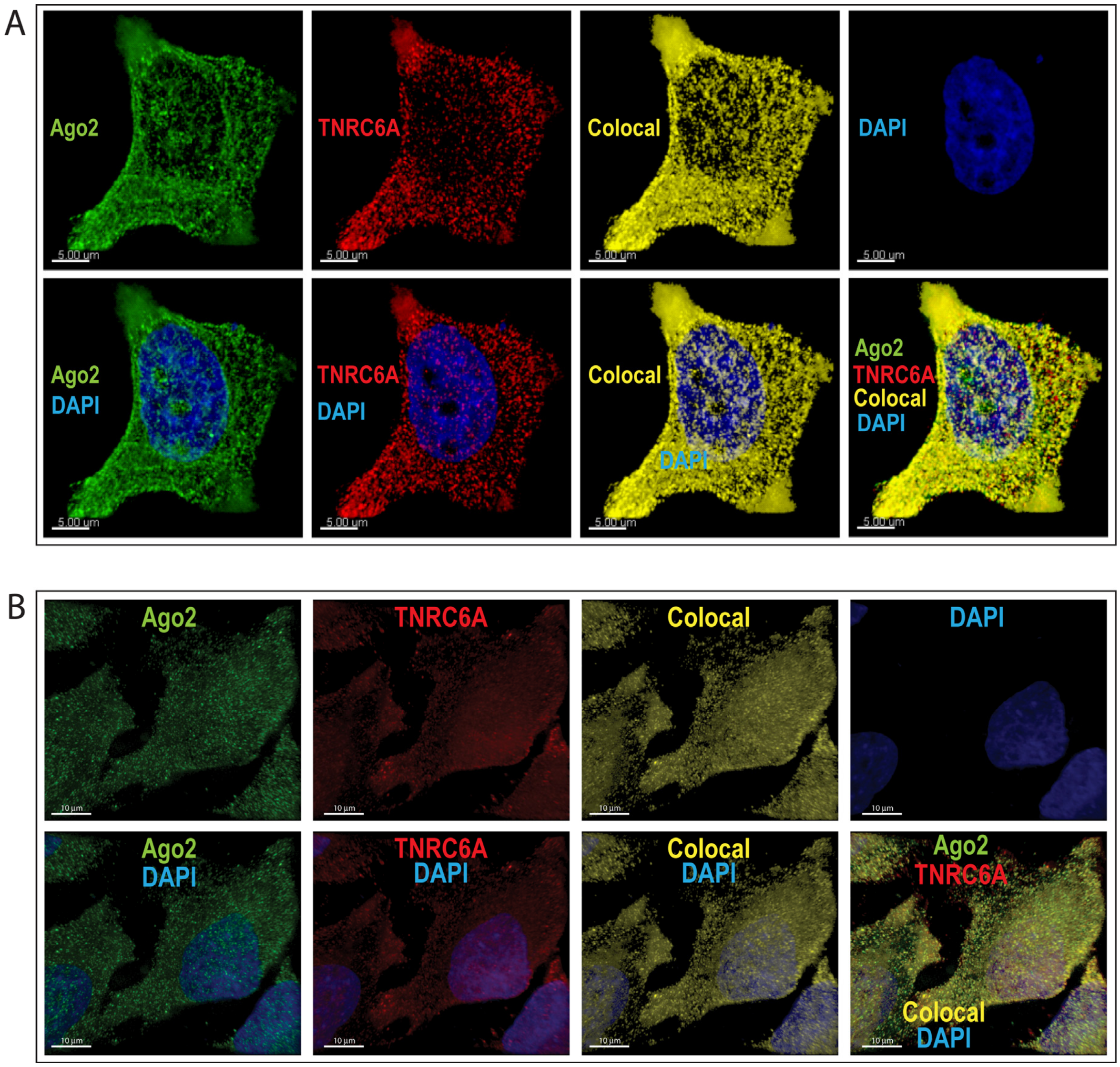

Figure S3, Related to Figure 3: Ago2 and TNRC6A immuno-staining co-localizes in HeLa cell nuclei. Cells were stained with anti-Ago2 from Abcam (**A**) or anti-Ago2 from Wako Chemicals (**B**) and anti-TNRC6A from Bethyl Laboratories (see Methods). (**A**) 4 µm thick and (**B**) 5 µm thick Z-stacks were taken. Co-localization channels were built in Imaris (Bitplane). Scale bars = 10 µm (**A**) and 5 µm (**B**).

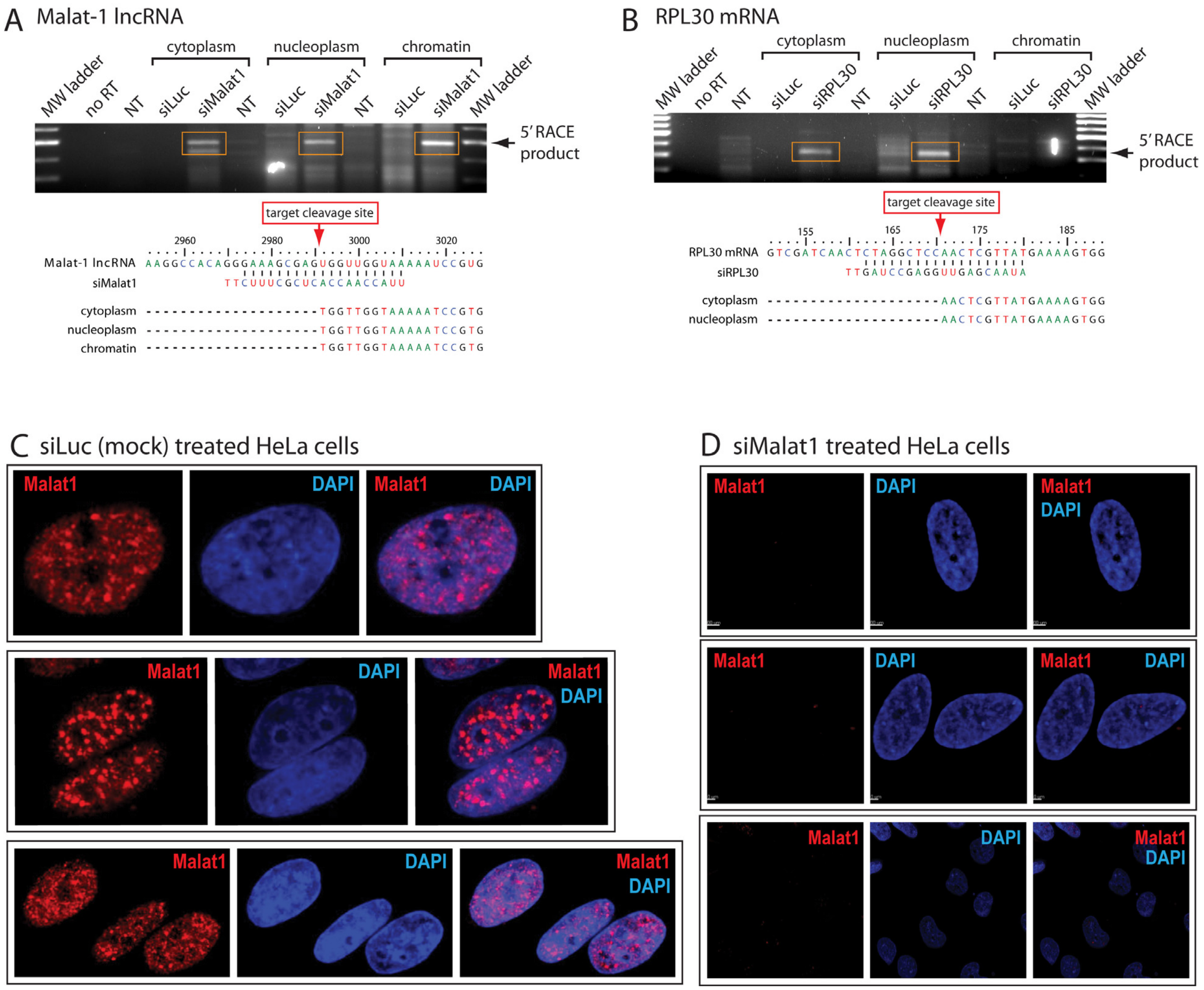

<u>Figure S4, Related to Figure 4</u>: 5'-RACE and Malat-1 FISH demonstrate siRNA-directed cleavage in nuclear fractions. HeLa cells were transfected with siRNA against Malat-1 lncRNA, RPL30 mRNA, with a negative control siRNA (siLuc) or left untreated. RNA from cytoplasmic, nucleoplasmic and chromatin fractions were isolated 72 h post-transfection and subjected to 5'RACE to detect predicted cleavage products for (**A**) Malat-1 or (**B**) RPL30 (see Methods). Gels for separation of RACE products are shown with the cleavage product band highlighted. Cleavage product bands were excised and sequenced to unambiguously validate sequence-specific siRNA-directed cleavage at the expected phosphodiester bond of the targeted RNA. 5' ends of sequenced RACE products are shown below each gel for each cell fraction. RACE was performed at least twice and cloning and sequencing performed several times per band for each experiment. HeLa cells were treated with (**C**) a mock siRNA (siLuc) or (**D**) siRNA against Malat-1 (siMalat1) then processed for FISH analysis (see Methods) 48 h later. Z-stacks 6 μm thick were taken.

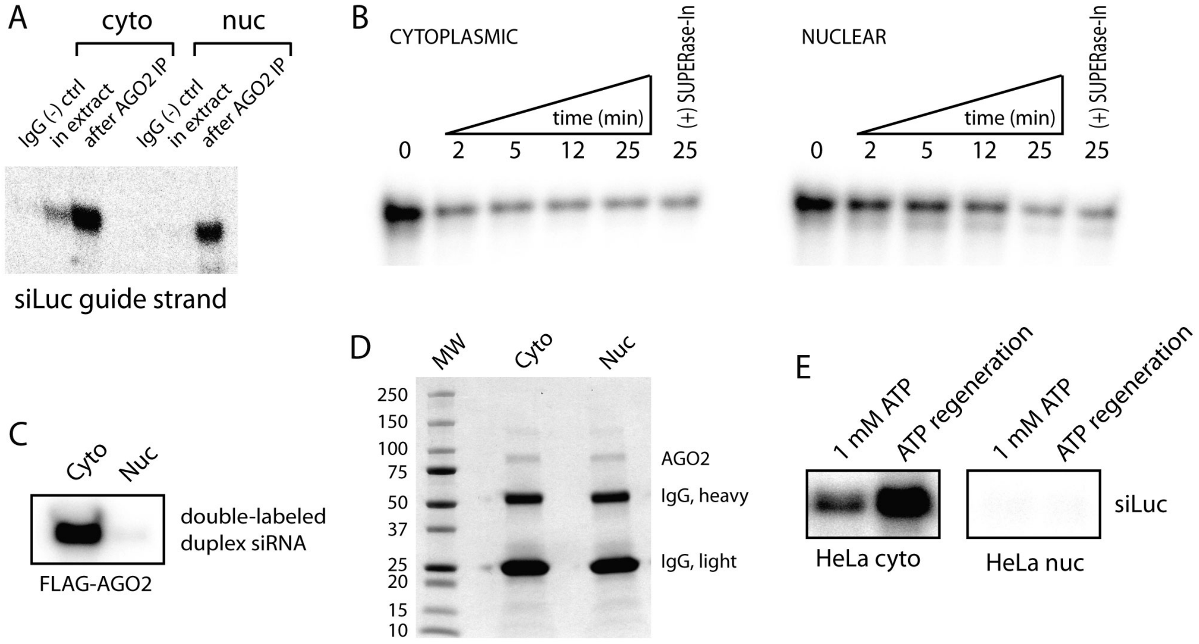

<u>Figure S5, Related to Figure 6</u>: Controls for Ago2 loading deficiency in the human cell nucleus. (**A**) Incubation of single-stranded siLuc guide RNA with T47D cytoplasmic and nuclear extracts followed by Ago2 immunoprecipitation, or with Ago2 after immunoprecipitation. Single-stranded guide RNA does not efficiently bind Ago2 in extracts presumably due to rapid degradation. If Ago2 is first immunoprecipitated to wash away contaminating nucleases, single-stranded guide RNA readily binds and co-immunoprecipitates. (**B**) Degradation kinetics of duplex siLuc in HeLa cytoplasmic or nuclear extracts. (+) SUPERase-In = addition of 50 unites of SUPERase-In RNase inhibitor. (**C**) Ago2 in vitro loading assay using siLuc siRNA dual-labeled on both guide and passenger strands. (**D**) Immunoprecipitation of Ago2 from HeLa cytoplasmic and nuclear extracts followed by coomassie staining shows equivalent amounts of Ago2 capture from both extracts. (**E**) Ago2 in vitro loading assay using radiolabeled siLuc and the addition of ATP regeneration system (see *Extended Methods*) in cytoplasmic or nuclear extracts.
