# Supplementary material for: RNAi Factors are Present and Active in Human Cell Nuclei
Source: Cell Rep. Author manuscript; Available in PMC 2015 Jan 16. (PMC3916906; doi:10.1016/j.celrep.2013.12.013)
Supplement: 03 [file NIHMS550334-supplement-03.pdf]

Table S1, Related to Figures 4 and 6: Small RNAs, primers, probes and oligonucleotides (5' to 3')

siRNAs/miRNAs		
name	sequence	notes
siLuc_as	rUrGrUrUrCrArCrCrUrCrGrArUrArUrGrUrGrCTT	antisense guide strand
siLuc_ss	rGrCrArCrArUrArUrCrGrArGrGrUrGrArArCrATT	sense passenger strand
siLuc_mm_as	rUrGrUrUrCrArCrCrUrGrCrArUrArUrGrUrGrCTT	antisense guide strand
siLuc_mm_ss	rGrCrArCrArUrArUrGrCrArGrGrUrGrArArCrATT	sense passenger strand
siLuc_mm3,6_ss	rGrCrUrCrArArArUrCrGrArGrGrUrGrArArCrATT	sense passenger strand
siLuc_mm9,10_ss	rGrCrArCrArUrArUrCrGrArGrGrUrGrArArCrATT	sense passenger strand
siLuc_mm13,16_ss	rGrCrArCrArUrArUrCrGrArGrCrUrGrUrArCrATT	sense passenger strand
has-miR-19a_5p	rArGrUrUrUrUrGrCrArUrArGrUrUrGrCrArCrUrArCrA	antisense guide strand
has-miR-19a_3p	rUrGrUrGrCrArArArUrCrUrArUrGrCrArArArArCrUrGrA	sense passenger strand
siMalat_as	rUrUrArCrCrArArCrCrArCrUrCrGrCrUrUrUrCTT	antisense guide strand
siMalat_ss	rGrArArArGrCrGrArGrUrGrGrUrUrGrGrUrArATT	sense passenger strand
siNeat_as	rUrUrUrCrUrArArGrCrArArCrUrUrCrUrCrArCTT	antisense guide strand
siNeat_ss	rGrUrGrArGrArArGrUrUrGrCrUrUrArGrArArATT	sense passenger strand
siRPL30_as	rArUrArArCrGrArGrUrUrGrGrArGrCrCrUrArGTT	antisense guide strand
siRPL30_ss	rCrUrArGrGrCrUrCrCrArArCrUrCrGrUrUrArUTT	sense passenger strand
siPPIA_as	rUrUrArGrGrArUrGrArArGrUrUrCrUrCrArUrCTT	antisense guide strand
siPPIA_ss	rGrArUrGrArGrArArCrUrUrCrArUrCrCrUrArATT	sense passenger strand
qPCR		
name	sequence	notes
Malat1 Prime Time Assay F	ACCATCGTTACCTTGAAACCG	forward primer for Malat-1 IDT prime time assay
Malat1 Prime Time Assay R	GATCTAGCACAGACCCTTCAC	reverse primer for Malat-1 IDT prime time assay
Malat1 Prime Time Assay P	/56-FAM/CTCACCTCG/ZEN/ATGCAGCCAGTAGC/3IABkFQ/	probe for Malat-1 IDT prime time assay
Neat1 Prime Time Assay F	TCTCTTCCTCCACCATTACCA	forward primer for Neat-1 IDT prime time assay
Neat1 Prime Time Assay R	CCTCCCTTTAACTTATCCATTCAC	reverse primer for Neat-1 IDT prime time assay
Neat1 Prime Time Assay P	/56-FAM/AACAATACC/ZEN/GACTCCAACAGCCACT/3IABkFQ/	probe for Neat-1 IDT prime time assay
RPL30 Prime Time Assay F	CACCAGTTTTAGCCAACATAGC	forward primer for RPL30 IDT prime time assay
RPL30 Prime Time Assay R	GATCAGACAAGGCAAAGCGA	reverse primer for RPL30 IDT prime time assay
RPL30 Prime Time Assay P	/56-FAM/ACAACTGCC/ZEN/CAGCTTTGAGGAAATCT/3IABkFQ/	probe forRPL30 IDT prime time assay
GAPDH Taqman Assay	proprietary	Applied Biosystems (Hs99999905_m1)
PPIA Taqman Assay	proprietary	Applied Biosystems (Hs99999904_m1)
5' RACE		
name	sequence	notes
GeneRacer RNA Adaptor	rCrGrArCrUrGrGrArGrCrArCrGrArGrGrArCrArCrUrGrA rCrArUrGrGrArCrUrGrArArGrGrArGrUrArGrArArA	5' RACE ligation adaptor
GeneRacer™ 5′ Primer	CGACTGGAGCACGAGGACACTGA	universal forward primer for 5' RACE PCR
GeneRacer™ 5′ Nested Primer	GGACACTGACATGGACTGAAGGAGTA	nested forward primer for 5' RACE PCR
MALAT15RACE1	CCCCCGCCTCAGTTACACATCCA	Malat-1-specific reverse primer for 5' RACE PCR
MALAT15RACE2	CCCCCGCCTCAGTTACACATCCAAA	Malat-1-specific reverse primer for 5' RACE PCR
RPL305RACE	CCGCATGCTGTGCCCAGTTCAAT	RPL30-specific reverse primer for 5' RACE PCR
RPL30-329-76	TCACCAGTCTGTTCTGGCATGCTTC	RPL30-specific reverse primer for 5' RACE nested PCR
RPL30nested-281-78	CAGAGTCACCTGGATCAATGATAGCC	RPL30-specific reverse primer for 5' RACE nested PCR
RPL30nested-202-78	GCCACTGTAGTGATGGACACCAGTTT	RPL30-specific reverse primer for 5' RACE nested PCR
RPL30nested-314-78	CACCAGTCTGTTCTGGCATGCTTCTA	RPL30-specific reverse primer for 5' RACE nested PCR

rN = RNA base; /56-FAM/ = 5' 6-FAM (Fluorescein); /ZEN/ = internal ZEN quencher; /3IABkFQ/ = 3' Iowa Black FQ quencher
